# Norms emerge through iterated learning

**DOI:** 10.1073/pnas.2504178122

**Published:** 2025-07-18

**Authors:** Scott Partington, Rachana Kamtekar, Shaun Nichols

**Affiliations:** ^a^Department of History and Philosophy of Science, University of Cambridge, Cambridge, UK CB2 3RH; ^b^Department of Philosophy, Cornell University, Ithaca, NY 14853

**Keywords:** norms, moral psychology, cultural evolution, iterated learning

## Abstract

Injunctive norms are universal: Every culture has rules that specify what actions are forbidden, obligatory, or permitted. Where do all of these norms come from? In this paper, we identify a mechanism of cultural transmission that can explain the emergence of injunctive norms. In particular, we develop an *iterated learning* account that shows how weak biases in pedagogy and inference can drive norm emergence through cultural transmission. Using transmission chain studies, we confirm the core predictions of the iterated learning account. In five studies (*N* = 3,688), an initial generation of participants learn about an action that is merely *inadvisable* and teach a new generation of participants about that action. After this learning process repeats iteratively, participants in later generations reliably judge that the action is *impermissible* and subject to punishment. We find this *inadvisable*-to-*impermissible* effect is elicited across a wide range of initial conditions. Overall, then, our results support the idea that iterated learning can drive the emergence of injunctive norms across a wide range of contexts.

## Significance

Across cultures, *norms* govern activities that range from the flow of traffic, dress codes, and table manners to moral rules about lying, harm, and theft. A large body of research has investigated the cognitive processes that enable people to learn and enforce norms. But before a norm can be learned or enforced, it must first exist. In this paper, we develop and test an account of how injunctive norms—rules about what people *ought* to do—can emerge through cultural transmission. Our studies show how a group that initially regards an action as *inadvisable* can, over generations, come to regard it as *impermissible* and subject to punishment. This demonstrates one way that injunctive norms can gradually emerge across cultures.

Every culture has *injunctive norms*, or rules about what is forbidden, obligatory, or permissible (1, 2). These norms ascribe actions or events with a *deontic status* (3, 4). This status marks norm violations as prohibited (e.g., lying, cheating, or stealing), rather than merely uncommon (e.g., walking backward), unfortunate (e.g., tripping over a rock), or imprudent (e.g., walking backward over the rock again). To thrive as we humans do—as interdependent, obligate cooperators—one must grasp these distinctions (5). While uncommon actions may lead to innovation, prohibited actions are often subject to punishment. Knowing the difference is critical.

Why are injunctive norms so common across cultures? Standard psychological accounts highlight various benefits of rules: They are easy to remember (6), promote cooperation (7), and often resonate with people’s antecedent emotions and goals (8). Moreover, these benefits can compound over time: Groups who cooperate more efficiently will do better than groups who do not (9). This dynamic explains why rule-based norms prevail and proliferate in culture. Yet for norms to confer these benefits, they must exist in the first place. What, then, leads to the *emergence* of new norms? To understand this, we must investigate the processes that create new norms, not just those which sustain preexisting norms.

In this paper, we identify a pathway to norm emergence. In doing so, we draw from recent research at the intersection of cognitive science and cultural evolution on *iterated learning* (10–20). This research emphasizes how people learn cultural items (e.g., a written language) from observing others, who also learned them from observing others, and so on and so forth, creating a chain of cultural transmission. Over generations, cognitive biases in teaching and learning can gradually reshape these items. Importantly, such biases do not have to be especially strong, nor innate; even weak biases can drive the emergence of cultural universals via iterated learning (21–25). Thus, iterated learning offers a mechanism by which small biases accumulate over time, allowing norms to emerge gradually without dramatic shifts in individual judgment. Crucially, this process does not require deference to a central authority—instead, norms can emerge through repeated, population-wide transmission.

Iterated learning accounts begin with a hypothesis about cognitive biases that could plausibly shape a cultural item. Next, one conducts *transmission chain studies* to test whether such processes can produce the target phenomenon. In these studies, participants in a “Generation 1” transmit a cultural item to participants in a “Generation 2,” who transmit that item to a “Generation 3,” and so on. For nearly a century (26), such study designs have been used to trace how information is transformed as it is repeated or spreads through social groups (27), often revealing biases in memory and communication (28–34). In what follows, we develop an iterated learning account of norm emergence and confirm its core predictions across a series of transmission chain studies.

First, though, it is important to emphasize that our account targets the emergence of *injunctive norms*—rules about what is allowed or not allowed in a given context. These are often contrasted with *descriptive norms*, which track what is common or uncommon (35). While descriptive norms can be inferred directly from others’ behavior, injunctive norms cannot. The leap from “is” to “ought”—from descriptive fact to deontic status—is a long-standing topic of interest in moral psychology (36), especially in recent work on norm acquisition (37) and normative judgment (38). Our account explains how a shift from “is” to “ought” can occur through cultural transmission.

## Norm Emergence from Iterated Learning

Our account posits two cognitive biases, in brief:

1. **Biased pedagogy.** In practical contexts, knowledgeable individuals (“teachers”) are biased toward communicating *relevant* yet *concise* information to naïve individuals (“learners”). Sometimes, the most relevant yet concise information about a candidate action *A* is simply “Don’t do *A*” or “You should not do *A*.”
2. **Deontic inference.** When teachers communicate information in the form of “Don’t do *A*” or “You should not do *A*,” learners may infer that *A*-ing is impermissible.

We will use transmission chain studies to test our account’s main prediction: If these biases feature across episodes of iterated learning, then a population will come to regard *A*-ing as *impermissible*, rather than merely *inadvisable*. That is, participants will infer that *A*-ing is forbidden and subject to punishment, not just unsafe or not preferred.

To begin, we detail and motivate each hypothesized bias.

### Biased Pedagogy.

Some utterances *describe* the world (e.g., “The berries are poisonous”), while others give *directives* about how to behave (e.g., “Don’t eat the berries!). Of course, speakers will often combine both, as in, “Don’t eat the berries because they are poisonous.” Sometimes, though, speakers give *bare directive* utterances without descriptive information. Why does this happen?

We suspect there is a domain-general principle underlying this tendency. Cognitive systems face a trade off between *reward* and *complexity* (39): How to achieve maximal value while expending minimal resources? (e.g., time, memory, etc.). This trade-off features in pedagogy whenever teaching has to be *relevant* yet *concise*. Here, we understand *relevance* in terms of decision-theoretic utility: A teacher’s utterance is relevant if it improves the learner’s decision-making (40). We understand *concision* in terms of the transmission cost of sending a message from teacher to learner: Typically, longer utterances have a greater transmission cost.

For a concrete example, consider these candidate utterances:

*Directive*: Don’t eat the berries.
*Descriptive*: The berries are poisonous.
*Directive*+*Descriptive*: Don’t eat the berries because the berries are poisonous.

All three options could deter berry consumption, yet they vary in length. *Directive*+*Descriptive* will likely offer the most relevance, but it is also the least concise. Thus, a teacher’s choice among these options depends on how they trade-off relevance and concision.

Say teachers in Generation 1 know the berries are poisonous, and they have to inform naïve learners about this. Even with a weak bias for concision, most teachers will transmit *Directive*+*Descriptive*. Across generations of iterated learning, however, a weak bias for concision is all that is needed for *Directive* utterances to proliferate in a cultural group. Consider how the subsequent generations of teachers will teach naïve learners. In Generation 2, many teachers retain the “ground truth” (i.e., the berries are poisonous), because they learned this information in Generation 1. Yet some of these teachers will only relay a bare *Directive*, due to their weak bias for concision. In addition, other teachers only received a bare *Directive* in Generation 1. These teachers lack any descriptive knowledge to pass on. Moreover, *Directive* is already relevant and maximally concise: “Don’t eat the berries” cannot be shortened without losing its meaning. Thus, this group of teachers can also be expected to relay *Directive* at high rates.

In particular, we suspect *Directive* will be favored over *Descriptive* for two main reasons. First, *Directive* plausibly has greater relevance than *Descriptive*, in so far as it directly conveys instructions on how to behave, whereas such advice must be inferred from *Descriptive*. Second, note that some teachers who received *Descriptive* as learners will relay *Directive* or a mixed summary, which may become a *Directive* in future generations. Yet teachers who receive *Directive* will not be able to teach *Descriptive*, because they do not know the underlying descriptive info. The overall consequence: Across generations of iterated learning, more and more teachers will communicate information in the form of *Directive*, relative to *Descriptive* or *Directive*+*Descriptive*.

Importantly, this outcome does not depend on the specific content of the descriptive information. Whether a candidate action is unsafe, unpleasant, or reputationally costly, bare directive summaries can facilitate optimal decisions for learners. Moreover, this kind of pedagogical instruction is common in parent–child interactions. Analysis of a large corpus of child-directed speech found that parents tend to give categorical injunctions when teaching young children how to behave (41): “Don’t hit anybody with that,” or “Tell him you’re sorry,” for example.

In summary, then, we hypothesize that when a candidate action is *inadvisable* and teachers have a weak bias for concision in pedagogy, bare directives are likely to proliferate via iterated learning. Indeed, prior work shows that cultural information tends to become more compressed over time (11–13), and we suspect the same tendency applies to pedagogical instructions about how to behave (42).

### Deontic Inference.

*Deontic inference* is the second cognitive bias in our account, describing learners’ tendency to infer deontic status from bare directives. Some bare directives convey advice to learners, but do not involve an action that is forbidden or subject to punishment, for example: “Don’t approach rattlesnakes.” Other bare directives plausibly communicate a deontic rule: “Don’t hit your brother,” for instance.

Now, consider an unfamiliar bare directive like “Don’t *dax*!” (43). Does “Don’t *dax*!” convey advice or a deontic rule? Since learners do not know the “ground truth” about *dax*-ing, the linguistic evidence is indeterminate—it could be mere advice, or it could express a deontic rule. Thus, learners have to make a judgment about how to interpret the evidence, perhaps by making an inference about which meaning is more likely. If learners are biased toward ascribing deontic status, they may infer that *dax*-ing is impermissible. In turn, these learners might give bare directives about *dax*-ing when teaching others, leading to the emergence of a deontic norm via iterated learning.

The same ambiguity can apply to “mixed” utterances with both directive and descriptive content, such as: “Don’t *dax* because *dax*-ing will injure others.” It is plausible that this utterance communicates both the deontic status of *dax*-ing, along with descriptive information about *dax*-ing. To be sure, the pragmatics here are complex and context-dependent: Factors such as tone, the relationship between the teacher and the learner, and perceived consensus can influence how these utterances are interpreted. But our main point is that mixed utterances can permit inferences about deontic status, all else being equal. If learners are biased toward inferring deontic status, when possible, they may infer *dax*-ing is impermissible even from mixed utterances.

Indeed, children infer deontic status in a wide range of contexts. For example, children infer deontic status based on adult demonstrations of intentional actions (43) and from behavioral regularities of social groups (37). Nativist accounts of norm psychology maintain that this reflects a specialized “norm acquisition mechanism” (2), where bias toward inferring deontic status is a key component. Alternatively, deontic inference may result from domain-general processing. Since deontic rules aid memory and coordination, resource-rational or reward-sensitive mechanisms may feature “inductive biases” that favor deontic inferences (6).

Regardless of the implementation, our prediction is this: *If* naïve learners are biased toward inferring deontic status from directive content, then novel injunctive norms can emerge in a cultural group via iterated learning.

### The Present Research.

To test our account’s key predictions, we conducted a series of transmission chain studies. Study 1 presents evidence that novel norms can emerge in an experimental population via iterated learning. Studies 2 to 5 investigate the scope and limits of this process. For each seed text—a vignette presented in Generation 1 (e.g., see Table 1)—we collected data across a four-generation transmission chain, with N = 90 participants in each generation (N = 360 per seed). Study 1 included five seed texts; Studies 2 to 5 each included one. After exclusions (*Materials and Methods*), the number of completed chains was *k*= 368 in Study 1, *k* = 75 in Study 2, *k* = 79 in Study 3, *k* = 79 in Study 4, and *k* = 71 in Study 5.

**Table 1.** Focal actions in Study 1

Scenario	Action	Seed text (presented in Generations 1 and 0)
All	–	On the Island of Cloud Hollow, there are two kinds of groups, “Hibbles,” and “Glerks.” Hibbles are blue creatures. Glerks are yellow. The island has different kinds of…
S1	Eat	…berries. It’s unsafe for Glerks to eat purple berries because purple berries are poisonous to Glerks.
S2	Swim	…rivers. It’s unsafe for Glerks to swim in purple rivers because purple water makes their blood freeze.
S3	Climb	…trees. It’s unsafe for Glerks to climb purple trees because the branches on the purple trees break easily.
S4	Sleep	…bushes. It’s unsafe for Glerks to sleep next to purple bushes because snakes live in purple bushes.
S5	Explore	…caves. It’s unsafe for Glerks to go into purple caves because tigers live in purple caves.

Across all studies, we used a *novel groups* paradigm in order to control for participants’ preexisting biases about human groups (37). Such designs are common in research on norm acquisition (44–46), and, despite their minimalism and simplicity, they often succeed in eliciting in-group biased judgments (47). This makes them a useful tool for testing how norms can emerge in the absence of entrenched group associations or social stereotypes.

## Study 1: The Inadvisable-to-Impermissible Effect

In Generation 1, participants read a short vignette introducing two groups, “Hibbles” and “Glerks.” They also learned about an action that was inadvisable for Glerks. We varied this focal action across five scenarios (Table 1). For example, in one scenario, participants learned: “It is unsafe for Glerks to eat purple berries because purple berries are poisonous to Glerks.”

Participants then received the following prompt: “Imagine you are a Glerk parent teaching your child about the island. What would you say?” After providing their summary, participants rated their agreement with several statements about the focal action (all 6-point scales; 1 = Strongly disagree, 6 = Strongly agree):

*Forbidden:* It is forbidden for Glerks to eat purple berries.
*Rule:* Glerks have a rule against eating purple berries.
*Permitted:* Glerks are permitted to eat purple berries.
*Allowed:* Glerks are allowed to eat purple berries.
*Trouble:* Glerks get in trouble for eating purple berries.
*Punish:* Glerks are punished for eating purple berries.
*Unsafe*: It is unsafe for Glerks to eat purple berries.

To establish a baseline for these judgments, we also collected data for a “Generation 0” where participants read the same text as Generation 1, but did not provide an open response. (Inclusion of Generation 0 does not affect the key statistical results).

In subsequent generations (Generations 2 to 4), each participant learned about the island from a summary that was randomly sampled (with replacement) from those produced by participants in the previous generation. After learning about the island, the study procedure was the same as Generation 1, with participants providing a summary and making the same series of judgments.

First, we coded participants’ summaries for directive expressions and descriptive facts (see *Materials and Methods* for coding instructions, exclusion criteria, and interrater reliability). Next, responses were coded as follows:

*Directive+Descriptive*: responses that relayed both a directive expression *and* a descriptive fact were given a score = 1; score = 0 otherwise.
*Bare Directive*: responses that relayed a directive expression, but not a descriptive fact, were given a score = 1; score = 0 otherwise.
*Bare Descriptive*: responses that relayed a descriptive fact, but not a directive expression, were given a score = 1; score = 0 otherwise.

In our statistical analyses, we analyzed the data using mixed-effects models that included a random intercept term for *Scenario*. We used a logistic regression model for the open response scores (*score* ∈ {0, 1}), and we used a linear regression model for the judgment ratings (*rating* ∈ {1, 2, 3, 4, 5, 6}). The models were fitted using the lme4 package in R, specified as follows:

**Open response**
  *score* ~ DV type * Generation + (1|Scenario)
**Judgment**
  rating ~ Generation + (1|Scenario)

The overall pattern of results was consistent across scenarios, so we report and discuss the combined results in the main text. We report scenario-specific results and supplementary analyses using ordinal logistic regression models in the *SI Appendix*; all confirm the findings presented in the main text.

## Results

### Open Response.

**Fig. 1.**
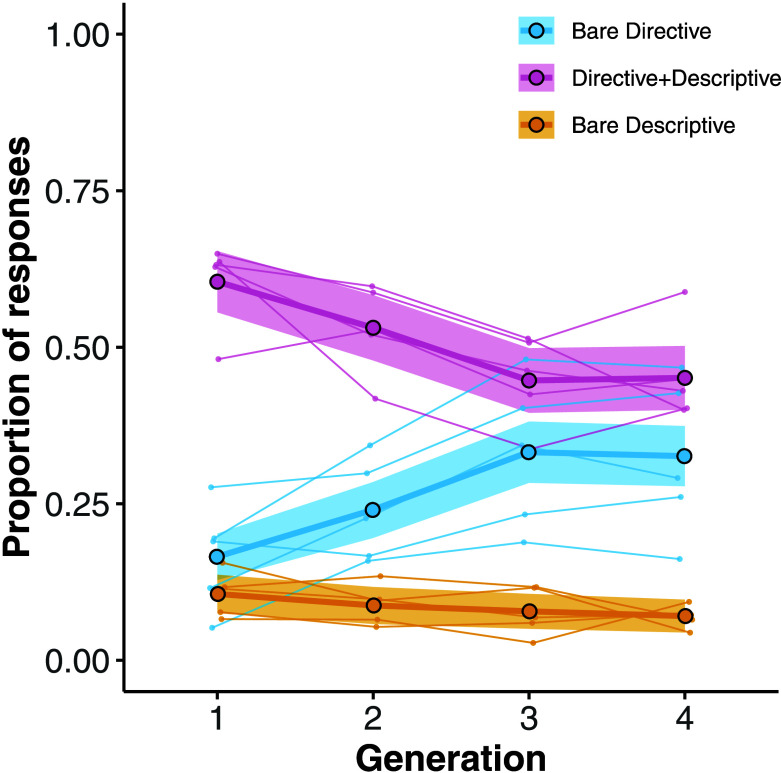
Study 1: Proportion of *Bare Directive* (blue), *Directive+Descriptive* (pink), and *Bare Descriptive* (yellow) responses (*y*-axis) across generations (*x*-axis). The thick lines with large dots correspond to the mean proportion across all five studies, with shaded regions corresponding to 95% CIs. The thin lines correspond to the mean proportion from the individual scenarios.

Across generations, the proportion of responses with directive content remained high, while the proportion of responses with descriptive content decreased (*b*_DV type × generation_ = −0.42, SE = 0.08, *P* < 0.001). This trend was primarily due to an increase in *Bare Directive* responses relative to both *Directive*+*Descriptive* responses (*b*_DV type × generation_ = −0.57, SE = 0.07, *P* < 0.001) and *Bare Descriptive* responses (*b*_DV type × generation_ = −0.50, SE = 0.07, *P* < 0.001) (Fig. 1). In later generations, participants more frequently gave responses of the form “Don’t eat the purple berries,” rather than responses that included descriptive information (e.g., “Don’t eat the purple berries because they are poisonous” or “Purple berries are poisonous”).

### Judgments.

**Fig. 2.**
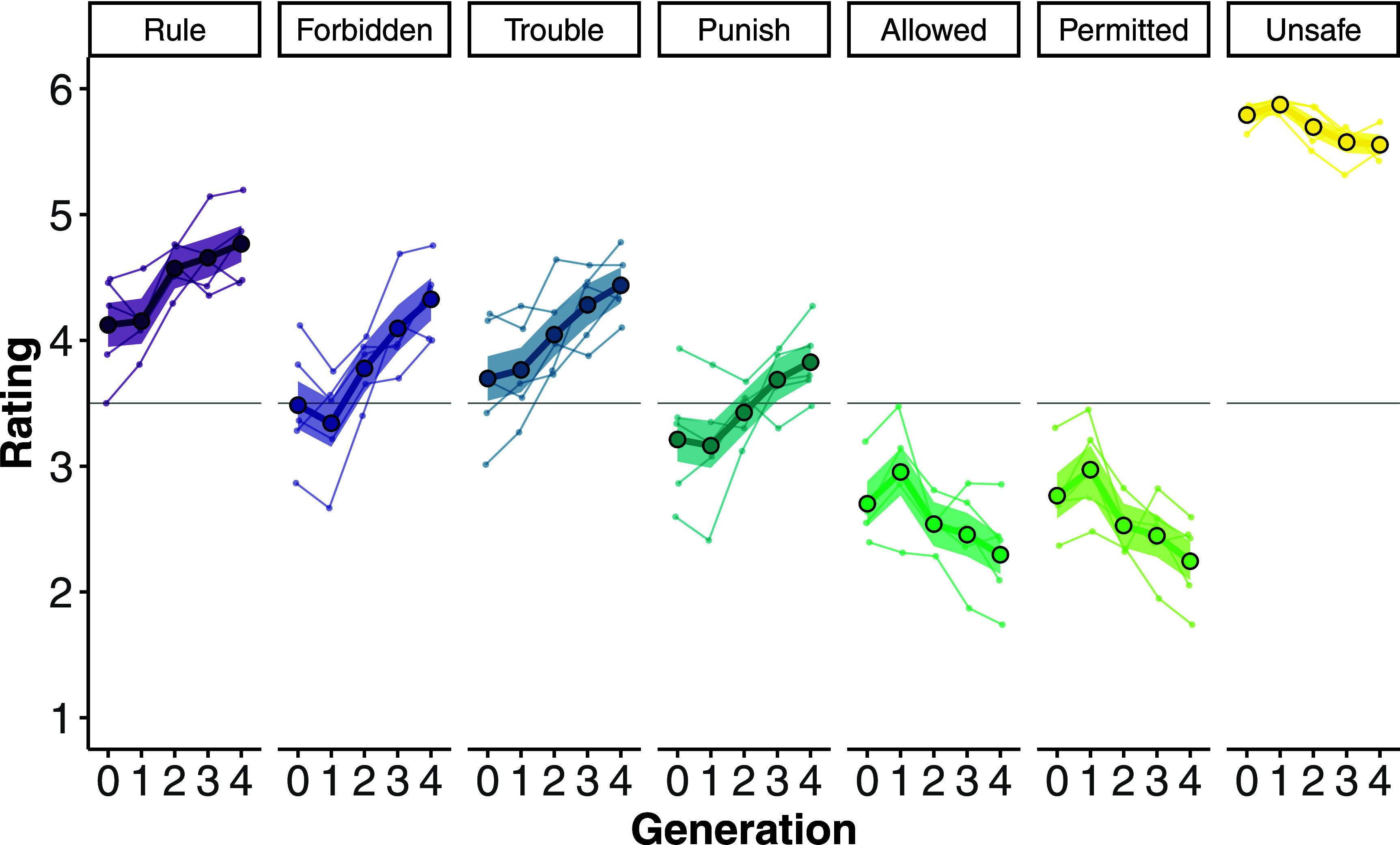
Study 1: Average scale rating (*y*-axis) across generations (*x*-axis) for each judgment measure (panels, from left to right: *Rule*, *Forbidden*, *Trouble*, *Punish*, *Allowed*, *Permitted*, *Unsafe*). Thick lines with large dots correspond to the mean rating across all five studies, with shaded regions corresponding to 95% CIs. Thin lines correspond to the mean rating from the individual scenarios. The horizontal line indicates the scale midpoint.

Across generations, ratings for prohibition judgment increased (*Rule*: *b* = 0.18, SE = 0.03, *P* < 0.001; *Forbidden*: *b* = 0.24, SE = 0.02, *P* < 0.001), and ratings for punishment judgments also increased (*Trouble*: *b* = 0.20, SE = 0.03, *P* < 0.001; *Punish*: *b* = 0.17, SE = 0.03, *P* < 0.001); by contrast, ratings for permission judgments decreased (*Allowed*: *b* = −0.13, SE = 0.03, *P* < 0.001; *Permitted*: *b* = −0.15, SE = 0.03, *P* < 0.001). Overall, then: across generations, participants increasingly judged that the focal action is prohibited and subject to punishment (Fig. 2).

The average ratings for *Forbidden* and *Punish* crossed the scale midpoint from Generation 0 to Generation 4 (*Forbidden*: *m*_Gen0_ = 3.48, 95% CI: [3.29, 3.67], *m*_Gen4_ = 4.32, 95% CI: [4.15, 4.49]; *Punish*: *m*_Gen0_ = 3.21, 95% CI: [3.04, 3.38], *m*_Gen4_ = 3.82, 95% CI: [3.67, 3.97]). So, on average, participants initially viewed the focal action as neither forbidden nor punished, yet, by Generation 4, participants came to view the focal action as both forbidden and punished. Apart from *Unsafe*, 95% CIs for all remaining judgments (*Rule*, *Trouble*, *Permitted*, *Allowed*) also crossed the scale midpoint in at least one study.

Ratings for *Unsafe* decreased across generations (*b* = −0.07, SE = 0.01, *P* < 0.001), though remained high overall (Fig. 2). This means that participants continued to regard the focal action as unsafe, despite a decline in the available descriptive information in the open responses.

## Discussion

Across five scenarios, we found that injunctive norms reliably emerged through iterated learning. In Generation 1, participants learned about an *inadvisable* action (e.g., eating poisonous berries), but did not receive any information about deontic status. By Generation 4, participants reliably inferred the focal action was *impermissible* and subject to punishment.

This *inadvisable*-to-*impermissible* effect was driven by two distinct processes. First, teachers’ pedagogy was biased: across generations, participants increasingly conveyed bare directives (e.g., “Don’t eat the purple berries!”), rather than providing descriptive information. Second, learners tended to infer deontic status from these bare directives. When combined through iterated learning, these processes suffice to explain the emergence of norms in our studies.

## Studies 2–5: The Scope of the Inadvisable-to-Impermissible Effect

So far, we have shown “existence proof” evidence for the inadvisable-to-impermissible effect. We have also shown the effect does not depend on a specific focal action (i.e., it is not an effect about eating berries). Still, many open questions remain. In a second series of transmission chain studies, we begin to investigate the scope of the effect.

First, we test whether the overall, multigeneration effect manifests across changes to the initial seed text, presented in Generation 1. To illustrate our approach, consider how the seed texts from Study 1 can be decomposed into various parts:

Seed text: …It is unsafe^1^ for Glerks^2^ to eat purple berries^3^ because purple berries are poisonous to Glerks^4^.

**Table:** 

Parts:	
*Evaluative status*	^1^It is unsafe…
*Subjects*	^2^for Glerks…
*Focal action*	^3^to eat purple berries…
*Descriptive information*	^4^because purple berries are poisonous to Glerks.

Across the five scenarios that we tested in Study 1, we varied the *focal action* and *descriptive information* while holding the *evaluative status* and *subject* parts constant. We found the inadvisable-to-impermissible effect was robust across these changes.

Next, we test whether the effect is robust across changes to evaluative status (Study 2) and subjects (Study 3). We also test whether judgments of impermissibility remain high when the focal action is originally assigned a deontic status (e.g., “Glerks are not allowed to eat purple berries”) and no further descriptive information is provided (Study 4); this is crucial to show that emergence is not easily reversed, or that “norm maintenance” through iterated learning is also robust. Last, in Study 5, we also test whether the inadvisable-to-impermissible effect occurs when the target of teaching is a naïve peer (“horizontal transmission”), rather than a child (“vertical transmission”).

### Study 2: Evaluative Status.

Using the same design and measures as in Study 1, Study 2 examined whether the inadvisable-to-impermissible effect occurs when the focal action is imprudent but not necessarily dangerous. We modified the seed text to read: “Glerks do not like eating purple berries because they think purple berries are icky.” Participants followed the same procedure as in Study 1, providing open responses and rating their agreement with the statements regarding the focal action.

As with Study 1, the proportion of *Descriptive* responses decreased relative to *Directive* responses across generations (*b*_DV type × generation_ = −0.48, SE = 0.16, *P* = 0.002). Again, this trend was due to an increase in *Bare Directive* responses, relative to *Directive+Descriptive* (*b*_DV type × generation_ = −0.60, SE = 0.17, *P* < 0.001) and *Bare Descriptive* responses (*b*_DV type × generation_ = −0.59, SE = 0.17, *P* < 0.001). Likewise, ratings for prohibition and punishment judgments increased across generations (*Rule*: *b* = 0.47, SE = 0.09, *P* < 0.001; *Forbidden*: *b* = 0.64, SE = 0.09, *P* < 0.001; *Trouble*: *b* = 0.60, SE = 0.08, *P* < 0.001; *Punish*: *b* = 0.52, SE = 0.07, *P* < 0.001), and ratings for permission judgments decreased across generations (*Permitted*: *b* = −0.56, SE = 0.09, *P* < 0.001; *Allowed*: *b* = −0.61, SE = 0.08, *P* < 0.001). Ratings for *Unsafe* also increased across generations (*b* = 0.64, SE = 0.09, *P* < 0.001), meaning that participants in later generations viewed eating berries as unsafe, rather than merely “icky” (*m*_Gen1_ = 2.68, SD = 1.65; *m*_Gen4_ = 4.62; SD = 1.74).

**Fig. 3.**
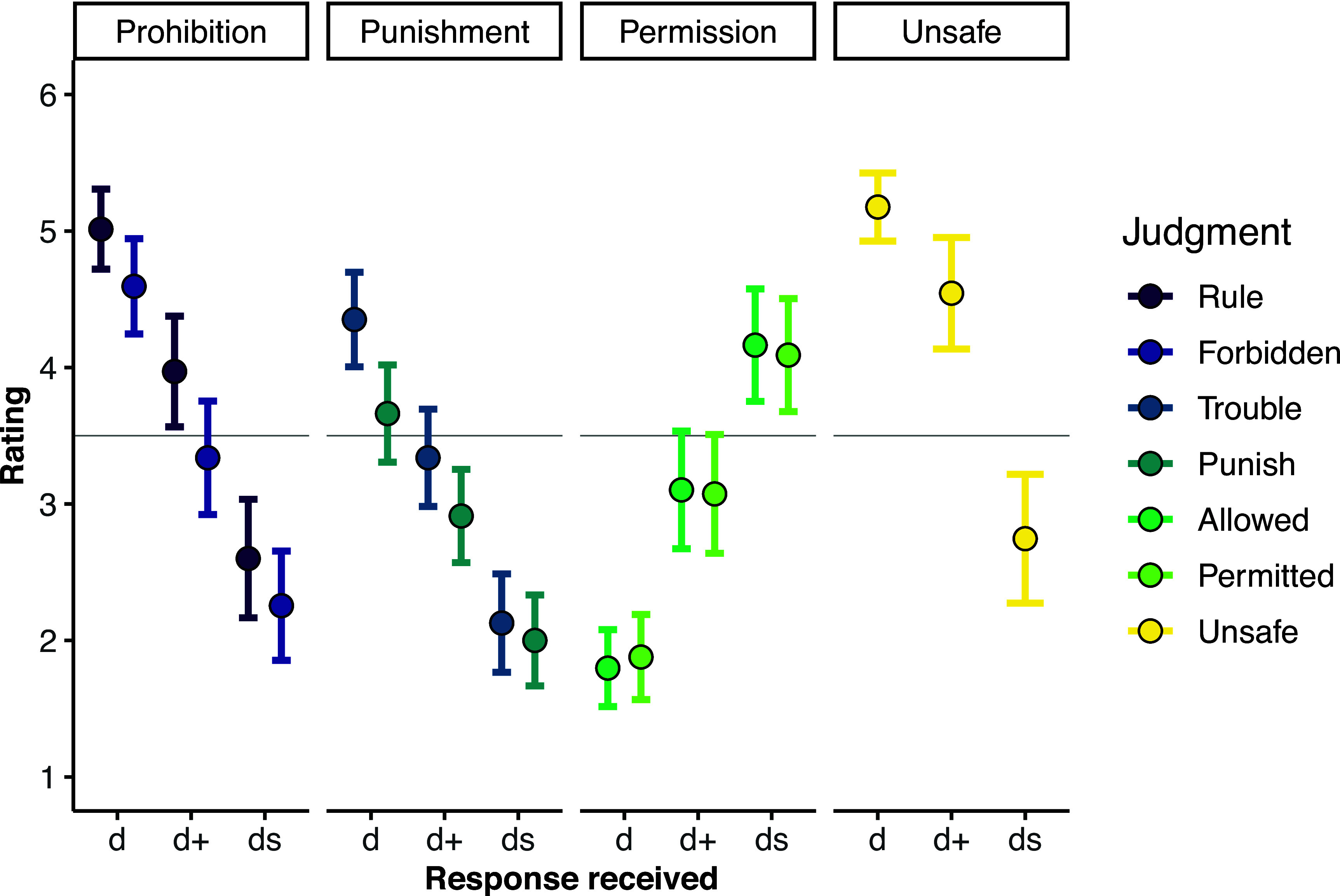
Study 2: Average scale rating (*y*-axis) by response type received (*x*-axis; *Bare Directive =* “d,”*Directive+Descriptive* = “d+,” *Bare Descriptive* = “ds”) for each judgment measure. Error bars correspond to 95% CIs. The horizontal line indicates the scale midpoint.

Since a plurality of participants expressed a *Bare Descriptive* response (e.g., *Bare Descriptive*: ${\hat{p}}_{\text{Gen}4}$ = 0.20; *Bare Directive*: ${\hat{p}}_{\text{Gen}4}$ = 0.39; *Directive+Descriptive*: ${\hat{p}}_{\text{Gen}4}$ = 0.32), we have the requisite data to analyze how participants’ judgments varied according to the type of response that they received. This analysis showed that *Bare Directive* responses were primarily responsible for changes in ratings across generations (Fig. 3). Compared to those who received a *Directive+Descriptive* response, participants who received a *Bare Directive* response were more likely to infer that eating berries was prohibited (*Rule*: *b* = 1.04, SE = 0.25, *P* < 0.001; *Forbidden*: *b* = 1.25, SE = 0.27, *P* < 0.001) and punished (*Trouble*: *b* = 1.01, SE = 0.25, *P* < 0.001; *Punish*: *b* = 0.75, SE = 0.25, *P* = 0.003), and less likely to infer that eating berries was permitted (*Permitted*: *b* = −1.20, SE = 0.27, *P* < 0.001; *Allowed*: *b* = −1.31, SE = 0.25, *P* < 0.001).

Overall, the results showed that norm emergence from iterated learning does not require that the focal action is initially regarded as unsafe; norm emergence can also occur when the action is initially viewed as safe, but unpleasant.

### Study 3: Subjects.

Study 3 investigated whether group identity markers inhibit norm emergence through iterated learning. The seed text was altered to indicate that it was unsafe for *Hibbles* rather than Glerks to eat purple berries. Participants were asked to summarize this information from the perspective of a Glerk teaching their child. Open responses were coded to distinguish between norms that treat Glerks as *subjects* (e.g., “Don’t eat the purple berries”) and norms that treat Hibbles as *patients* (e.g., “Don’t give purple berries to Hibbles”).

In Generation 1, few participants relayed a *Bare Directive* response for either a patient norm (${\hat{p}}_{\text{Gen}1}$ = 0.04) or a subject norm (${\hat{p}}_{\text{Gen}1}$ = 0.09). Across generations, however, a subject norm emerged: The proportion of responses expressing a *Bare Directive* about Glerks as subjects increased relative to responses expressing a *Bare Directive* about Hibbles as patients (*b*_DV type × generation_ = 0.85, SE = 0.30, *P* = 0.004). By Generation 4, a plurality of participants expressed a *Bare Directive* about Glerks as subjects (${\hat{p}}_{\text{Gen}4}$ = 0.44), whereas the proportion of *Bare Directive* responses about Hibbles as patients remained very low (${\hat{p}}_{\text{Gen}4}$ = 0.03).

The judgment measures show a similar pattern of results. In Generation 1, participants gave low ratings for prohibition (*Rule*: *m =* 1.43, SD = 0.86; *Forbidden*: *m* = 1.39, SD = 0.80) and punishment judgments (*Trouble*: *m =* 1.51, SD = 1.09; *Punish*: *m* = 1.48, SD = 0.94), and participants gave high ratings for permission judgments (*Allowed*: *m* = 5.19, SD = 1.16; *Permitted*: *m* = 5.18, SD = 1.10). Across generations, though, ratings for prohibition and punishment judgments increased (*Rule*: *b* = 0.69, SE = 0.09, *P* = 0.007; *Forbidden*: *b* = 0.63, SE = 0.09, *P* = 0.008; *Trouble*: *b* = 0.60, SE = 0.09, *P* < 0.001; *Punish*: *b* = 0.48, SE = 0.08, *P* < 0.001) and ratings for permission judgments decreased (*Permitted*: *b* = −0.57, SE = 0.10, *P* < 0.001; *Allowed*: *b* = −0.58, SE = 0.10, *P* < 0.001). *Unsafe* ratings increased across generations (*b =* 0.78, SE = 0.10, *P* < 0.001).

In summary, participants in Generation 1 learned about an action that was unsafe for Hibbles. Across generations, however, participants increasingly viewed the same action as prohibited, punished, not permitted, and unsafe *for Glerks*. Hence the core processes that we have investigated are not necessarily constrained by markers of group identity, as emphasized in prior accounts of norm psychology (48). This result raises the possibility that iterated learning can lead to the emergence of norms that apply across group boundaries.

### Study 4: Norm Maintenance.

Study 4 tested whether injunctive norms are maintained through iterated learning once they have emerged. Participants in Generation 1 were presented with bare directive information: “Glerks are not allowed to eat purple berries.” We predicted that rates of bare directive responses would remain high across generations, thereby preserving the norm’s deontic status.

**Fig. 4.**
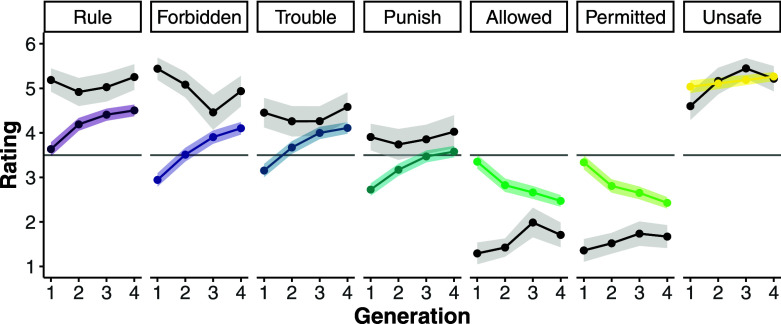
Studies 1–5: Across generations (*x*-axis), average scale rating (*y*-axis) for each judgment (panels). Black lines and dots correspond to results from Study 4. Colored lines and dots correspond to average ratings from all other studies. The shaded region corresponds to 95% CIs. The horizontal line indicates the scale mid-point.

The results confirmed these predictions. Across generations, the proportion of bare directive responses remained high (${\hat{p}}_{\text{Gen}1}$ = 0.91; ${\hat{p}}_{\text{Gen}2}$ = 0.75; ${\hat{p}}_{\text{Gen}3}$ = 0.70; ${\hat{p}}_{\text{Gen}4}$ = 0.63) and the proportion of bare descriptive responses remained low (${\hat{p}}_{\text{Gen}1}$ = 0.01; ${\hat{p}}_{\text{Gen}2}$ = 0.05; ${\hat{p}}_{\text{Gen}3}$ = 0.09; ${\hat{p}}_{\text{Gen}4}$ = 0.03). Accordingly, prohibition and punishment judgments remained high (in Generation 4, *Rule*: *m* = 5.25, SD = 1.29; *Forbidden*: *m* = 4.93, SD = 1.54; *Trouble*: *m* = 4.58, SD = 1.46; *Forbidden*: *m* = 4.03, SD = 1.66), and permission judgments remained low (in Generation 4, *Allowed*: *m* = 1.70, SD = 1.24; *Permitted*: *m* = 1.67, SD = 1.15). The population’s views about deontic status remained constant overall (Fig. 4). *Forbidden* (b = −0.21, SE = 0.07, *P* = 0.005), *Permitted* (b = 0.11, SE = 0.06, *P* = 0.047), and *Allowed* (b = 0.17, SE = 0.06, *P* = 0.004) ratings showed a statistical difference across generations, though none crossed the scale midpoint.

Briefly, it is also worth highlighting that the proportion of *Directive+Descriptive* responses increased across generations (*b* = 0.51, SE = 0.16, *P* = 0.001). In tandem, *Unsafe* ratings also increased across generations (*b =* 0.21, SE = 0.06, *P* = 0.001). So, a plurality of participants made a reverse inference: Having learned that eating purple berries was not allowed, they inferred that eating purple berries is unsafe and opted to transmit this information to learners. In this way, the rise in *Directive*+*Descriptive* responses across generations likely contributed to the observed shift in judgment ratings: As *Bare Directive* responses became less common, participants were less likely to infer the focal action is forbidden and more likely to infer it is allowed.

### Study 5: Horizontal Transmission.

In Studies 1–4, we asked participants to assume the role of a Glerk parent teaching a child. In effect, then, our studies modeled a *vertical* transmission process, where information is transferred from a preceding generation to a subsequent generation. But cultural transmission occurs within generations, too. Moreover, our account predicts that biased pedagogy occurs when teaching naïve learners, not children per se. So, our account predicts that norm emergence through iterated learning can also occur in the context of peer-to-peer, *horizontal* transmission, so long as one is teaching a *naïve* peer. In Study 5, we test this prediction by asking participants, “Imagine you are a Glerk, and you’re meeting a new Glerk who has just moved to the island. What would you say to teach the new Glerk about the island?”

Results remained consistent with the iterated learning account. Across generations, the proportion of *Descriptive* responses decreased relative to *Directive* responses (*b* = −0.51, SE = 0.16, *P* = 0.001). As with previous studies, this was attributable to an increase in *Bare Directive* responses relative to *Directive+Descriptive* (*b* = −1.00, SE = 0.16, *P* < 0.001) and *Bare Descriptive* (*b* = −0.61, SE = 0.21, *P* = 0.004) responses. Across generations, participants’ ratings for prohibition judgments and punishment judgments increased (*Rule*: *b* = 0.24, SE = 0.09, *P* = 0.007; *Forbidden*: *b* = 0.26, SE = 0.09, *P* = 0.008; *Trouble*: *b* = 0.32, SE = 0.09, *P* < 0.001; *Punish*: *b* = 0.25, *SE* = 0.09, *P* = 0.005). Ratings for permission judgments did not differ significantly across generations (*Permitted*: *b* = −0.13, SE = 0.09, *P* = 0.13; *Allowed*: *b* = −0.11, SE = 0.09, *P* = 0.21).

These findings show that norm emergence from iterated learning can occur not only in vertical transmission from parent to child, but also in horizontal transmission from peer to peer.

## General Discussion

In our studies, initial generations received no information about the deontic status of the focal action. Yet, later generations viewed the action as forbidden and expected that individuals are punished for taking that action. This effect was not specific to one action or another (e.g., eating poisonous berries), but rather occurred when decision-relevant yet concise utterances about an action *A* can take the form “Don’t do *A*” or “You shouldn’t do *A*.” Learners interpreted such directives as evidence that the action is impermissible, not merely inadvisable. These learners then transmitted bare directives to others, repeating the process iteratively as teachers. The result: Later generations reliably ascribed a deontic status to the focal action, even though initial generations had no information about the action’s deontic status to begin with.

Since the processes that drive this inadvisable-to-impermissible effect are highly general, it is plausible that biased pedagogy, deontic inference, and iterated learning can together generate novel norms across diverse cultural contexts. If so, the iterated learning account fills an important gap in the standard explanation of why injunctive norms are so common across cultures. The revised account would go like this: i) injunctive norms often *emerge* from iterated learning due to cognitive biases in pedagogy and inference, then ii) injunctive norms *persist* because rules often confer benefits for learning, memory, social coordination, and emotional resonance.

In general, the iterated learning framework offers a mechanism by which small biases can accumulate over time to produce substantial cultural change. This gradualism is a core strength of our account: It explains how norms can emerge incrementally, without dramatic shifts in individual judgment. It also shows how norms can arise without deference to a central authority—emerging instead through repeated, population-wide transmission. Even weak biases toward deontic inference can, over generations, give rise to novel norms.

Before we discuss further theoretical implications of this account, first we must address the limitations of the present research. So far, we have characterized biased pedagogy and deontic inference in a broad manner. While our initial findings support this broad account, many questions remain about how exactly these processes operate. A more precise account would further clarify when and why norms can emerge via iterated learning. For instance, peer disagreement, inconsistent messaging, or low perceived authority of the speaker may inhibit deontic inference and block norm emergence. Investigating such factors in depth is a valuable direction for future research, particularly for clarifying the boundary conditions on norm emergence through iterated learning. This is a substantial, worthy project in its own right, but it is beyond the scope of this initial paper.

Our findings are also limited in important ways. All participants were English-speaking adults from the United States, so cross-cultural samples are needed to provide comprehensive evidence. Developmental studies are also needed to assess when and to what extent children exhibit the biases that we posit here. Additionally, our study designs simplify the complexities of real-world norm emergence. For instance, in our studies, each transmission episode involves a teacher and a completely naïve learner who never have repeated interactions. We do not think this idealization threatens the main upshots of our account, but this is an empirical question that is worthy of further investigation. Future work could explore how repeated interactions—where trust, reputation, or shared history influence communication—might shape the emergence or stability of norms.

Further research could also examine factors such as opportunities for individual learning, social network structure, and how prosocial norms may emerge (e.g., “Help those in need”). Study 2 showed that norm emergence from iterated learning is not limited to actions involving physical harm. But it remains an open question whether prohibitions can also emerge in domains such as reputational damage, financial risk, or violations of group identity. Testing a broader range of seed texts will be key to assessing the generalizability of the present findings. Likewise, Study 3 showed that norm emergence from iterated learning is not necessarily constrained by group identity. However, since the present research used a novel groups paradigm it remains an open question whether similar effects would extend to real-world social categories.

Another question of interest is how the emergence of injunctive norms may relate to preexisting descriptive norms. Here, a potentially related phenomenon is children’s tendency to infer deontic status from generic statements about group behaviors. For example, when told that “Glerks eat purple berries and Hibbles eat orange berries,” many children infer that it is *not okay* for Glerks to eat purple berries (37). If parents tend to give children these kinds of generic statements, a “normal-to-impermissible” emergence pathway may also occur via iterated learning, too. In particular, our iterated learning account predicts that descriptive norms with some *prudential basis*—that help subjects avoid risk of harm, reputational costs, etc.—are especially likely to give rise to injunctive norms.

To conclude, we want to highlight a key theoretical upshot: The iterated learning account can explain why the functions of norms are often opaque to those who follow and enforce them (49). For example, in Western cultures, most of us do not know *why* we shake hands to greet each other, only that it is “the right thing to do.” But suppose that handshaking started out as a way to show that neither person is carrying a weapon. The iterated learning account would explain why this descriptive information was lost to history. Through biases in pedagogy and inference, teachers would instruct learners to shake hands using bare directives, which, over time, became entrenched and maintained via iterated learning. The result being, most of us know that shaking hands is the right thing to do, but we could not explain the norm’s original purpose.

In this way, iterated learning can explain why norms become opaque over time, but this opacity may leave them vulnerable to being overturned. In some cases, that is beneficial—such as when a norm’s original function is no longer relevant (e.g., food taboos in times of scarcity). In other cases, losing sight of a norm’s function can make it easier to dismantle an important norm, as when procedural conventions are eroded and democratic institutions weakened. In this way, the opacity created by iterated learning is a double-edged sword: It allows for normative change, but that change can be stabilizing or destabilizing.

The iterated learning dynamic may also help explain the emergence of more foundational norms. In many species, including humans, an individual who occupies territory will fight more aggressively than others to retain that occupancy. Given such territorialism, it is often imprudent to try to displace an occupant, and many species register this, displaying a kind of territorial deference (50). But of course we also think that it is typically *impermissible* to take territory occupied by another. One possibility is that the recognition of the imprudence of taking led, through cultural transmission, to the emergence of property norms.

In addition, the explanatory promise of this account might extend to situations with the apparent structure of prisoner’s dilemmas (51, 52). Defecting in cooperative activities can yield an immediate benefit, but it also carries reputational risks, suggesting that the individual is an unreliable partner (53). That provides a reason to teach kith and kin not to defect—it is in their long-term best interests. Crucially, though, cultural transmission does not just present defection as *inadvisable* but reshapes it as outright *impermissible*. In this way, the iterated learning account can help explain why cooperative behaviors are so often regarded as moral imperatives, rather than mere strategic choices (54).

## Materials and Methods

Study materials, data, and code are available on OSF. All studies were approved by the Institutional Review Board at Cornell University. All participants provided informed consent to take part in the online studies.

### Coding for Open Responses.

Across all studies (excluding Study 3; see main text), the coding criteria for open responses were as follows. First, we coded whether the response contained a directive expression:

*Directive*: responses that relayed an imperative (e.g., “Don’t eat the purple berries”) or normative guidance about the focal action (e.g., “You should never eat purple berries”) were given a score = 1; score = 0 otherwise.

In our coding criteria, we considered the following phrases as conveying normative guidance about an action *A*: “follow the rule about *A*-ing,” “have to *A*,” “need to *A*,” “must/must not *A*,” “should/should not *A*,” “supposed to/not supposed to *A*,” “allowed/not allowed to *A*,” “cannot A,” “only A-ing is okay,” and nonimperatival forms of “do not” (e.g., “We do not *A*” or “One does not *A*”). See *SI Appendix* for counts of each kind of normative guidance in Study 1.

Our analyses in the main text combined responses that gave imperatives and normative guidance, since both are consistent with—but do not necessitate—ascriptions of deontic status. The main results of our analyses also hold when imperatives and normative guidance are analyzed as distinct subcategories (*SI Appendix*). We also report additional summaries of response content in the *SI Appendix*.

Next, we coded whether the response relayed a descriptive fact:

*Descriptive*: responses that relayed a descriptive fact about the focal object (e.g., “Purple berries are poisonous”) were given a score = 1; score = 0 otherwise.

In particular, we coded for descriptive facts related to the “ground truth” in the seed text from Generation 1 (e.g., Study 1: “It is unsafe for Glerks to eat purple berries because purple berries are poisonous for Glerks.”).

Responses that did not mention the focal action from the seed text were excluded from analyses and not eligible for transmission to the next generation. The rates of such “low quality” responses were low overall (e.g., in Study 1: 7.7%, or 154 out of 2,013 responses were coded as low quality).

For each study, a large, randomly selected subset of responses were scored by two coders who did not know from which generation the responses originated. Interrater agreement was high (percent agreement: 94% (1,327/1,411), Cohen’s κ = 0.91) and remaining disagreements were resolved in discussion between coders.

### Inclusion Criteria.

Across all studies, we recruited N = 90 participants (Prolific, US) for each generation in the transmission chain. To be included in the participant pool, participants must i) have reported English as a fluent language, ii) have participated in at least 40 previous surveys on Prolific, iii) have been approved for compensation in at least 95% of those surveys, and iv) not previously participated in a study that was part of this line of research.

### Materials.

See *SI Appendix* for study-specific seed texts and judgment measures.

### Study 1.

#### Preregistration.

See OSF for a preregistered analysis plan.

#### Participants.

We recruited *N* = 2,240 participants. Participants who failed a simple attention check (*n* = 227) or provided an open response that did not mention the focal action (*n* = 154) were excluded from analysis. In total, *n* = 1,859 participants (*m*_age_ = 37.91 y, SD = 13.11 y) were included in the analyses.

#### Procedure.

Participants in Generation 1 were asked to summarize a seed text that mentioned a focal action that was unsafe for Glerks (see main text for details). Next, Participants made a series of judgments about the focal action (presented in randomized order; see main text for details). Participants in Generations 2–4 followed the same procedure, except they received and summarized a randomly sampled response from the preceding generation (sampled with replacement). This design reflects a situation in which each participant in Generation *n* + 1 has an equal chance of learning from any member of Generation *n*. Participants in Generation 0 provided judgment ratings but did not provide an open response.

### Study 2.

#### Participants.

We recruited N = 366 participants in total (Prolific, US; approx. N = 90 per generation). Participants who failed a simple attention check (*n* = 26) or provided an open response that did not mention the focal action (*n* = 45) were excluded from analysis. In total, *n* = 295 participants (*m*_age_ = 36.82 y, SD = 13.10 y) were included in the analyses.

#### Procedure.

The study procedure was identical as Study 1, except the seed text read that “Glerks do not like eating purple berries because they think purple berries are icky.”

### Study 3.

#### Participants.

We recruited N = 362 participants in total (Prolific, US; approx. N = 90 per generation). Participants who failed a simple attention check (*n* = 38) or provided an open response that did not mention the focal action (*n* = 39) were excluded from analysis. In total, *n* = 285 participants (*m*_age_ = 39.98 y, SD = 13.47 y) were included in the analyses.

#### Procedure.

The study procedure was identical as Study 1, except the seed text read that “It’s unsafe for Hibbles to eat purple berries because purple berries are poisonous to Hibbles.”

### Study 4.

#### Participants.

We recruited N = 359 participants in total (Prolific, US; approx. N = 90 per generation). Participants who failed a simple attention check (*n* = 23) or provided an open response that did not mention the focal action (*n* = 33) were excluded from analysis. In total, *n* = 303 participants (*m*_age_ = 34.50 y, SD = 12.16 y) were included in the analyses.

#### Procedure.

The study procedure was identical as Study 1, except the seed text read that “Glerks are not allowed to eat purple berries.”

### Study 5.

#### Participants.

We recruited N = 361 participants in total (Prolific, US; approx. N = 90 per generation). Participants who failed a simple attention check (*n* = 37) or provided an open response that did not mention the focal action (*n* = 27) were excluded from analysis. In total, *n* = 297 participants (*m*_age_ = 37.22 y, SD = 12.53 y) were included in the analyses.

#### Procedure.

The study procedure was identical as Study 1, except the summary instructions were “Imagine you are a Glerk, and you’re meeting a new Glerk who has just moved to the island. What would you say to teach the new Glerk about the island?”

## Supplementary Material

Appendix 01 (PDF)

## Data Availability

Study materials, data, code data have been deposited in OSF (https://osf.io/cznme/?view_only=da63448142ab4e05ba86504e2b550c8d) (55). All other data are included in the article and/or *SI Appendix*.
