# Supplementary material for: Norms emerge through iterated learning
Source: Proc Natl Acad Sci U S A. 2025 Jul 18;122(29):e2504178122. doi: 10.1073/pnas.2504178122 (PMC12304962; doi:10.1073/pnas.2504178122)
Supplement: Supplementary file 1 — Appendix 01 (PDF) [file pnas.2504178122.sapp.pdf]

Supporting Information for
Norms emerge through iterated learning

Scott Partington[1], Rachana Kamtekar[2], Shaun Nichols[2]
[1]Department of History and Philosophy of Science, University of Cambridge
[2]Department of Philosophy, Cornell University

*Corresponding author: Scott Partington
Email: sp933@cam.ac.uk

This PDF file includes:

Supporting text
Figures S1 to S11
Table S1

Supporting Information

Data availability. Data and analysis scripts are available at OSF.

Study 1: The inadvisable-to-impermissible effect

Materials. On the first screen of the survey, participants read an introduction text (see Fig. S1). In Generations 0-1, the introduction text read:

> *On the next screen, you will see a one paragraph story about creatures that live on a fictional island called "Hibbles" and "Glerks."*
>
> *It is very important that you read the information at a pace that allows you full comprehension as you will be asked to summarize this information later.*

In Generations 2-4, the introduction text read:

> *In a previous version of this study, we asked participants to summarize a short narrative about creatures that live on a fictional island called "Hibbles" and "Glerks."*
>
> *On the next screen, you will see a summary that was written by a previous participant from the perspective of a Glerk.*
>
> *It is very important that you read the information at a pace that allows you full comprehension as you will be asked to summarize this information later.*

Next, participants received either the study-specific seed text (for generation 1 only; full materials below) or a response that was randomly sampled (with replacement) from the set of responses given by participants in the previous generation (for generations 2-4). In each generation, participants received the same summary instructions:

> *Summary instructions (Study 1, generations 1-4)*:
> "Imagine you are a Glerk parent teaching your child about the island. What would you say?"

The study-specific materials were the following (see Fig. S2 for an example of judgment DV as viewed by participants):

Study 1 ("Eat")

Seed text (S1, generations 0-1)

On the Island of Cloud Hollow, there are two kinds of groups, "Hibbles," and "Glerks". Hibbles are blue creatures. Glerks are yellow. The island has different kinds of berries. It's unsafe for Glerks to eat purple berries because purple berries are poisonous to Glerks.

Judgment DVs (S1, all generations)

forbidden: It is forbidden for Glerks to eat purple berries.
rule: Glerks have a rule against eating purple berries.
permitted: Glerks are permitted to eat purple berries.
allowed: Glerks are allowed to eat purple berries.
trouble: Glerks get in trouble for eating purple berries.
punish: Glerks are punished for eating purple berries.
unsafe: It is unsafe for Glerks to eat purple berries.

Study 1 ("Swim")

Seed text (S2, generations 0-1)

On the Island of Cloud Hollow, there are two kinds of groups, “Hibbles,” and “Glerks”. Hibbles are blue creatures. Glerks are yellow. The island has different kinds of rivers. It’s unsafe for Glerks to swim in purple rivers because purple water makes their blood freeze.

Judgment DVs (S2, all generations)
forbidden: It is forbidden for Glerks to swim in purple rivers.
rule: Glerks have a rule against swimming in purple rivers.
permitted: Glerks are permitted to swim in purple rivers.
allowed: Glerks are allowed to swim in purple rivers.
trouble: Glerks get in trouble for swimming in purple rivers.
punish: Glerks are punished for swimming in purple rivers.
unsafe: It is unsafe for Glerks to swim in purple rivers.

Study 1 (“Climb”)
Seed text (S3, generations 0-1)
On the Island of Cloud Hollow, there are two kinds of groups, “Hibbles,” and “Glerks”. Hibbles are blue creatures. Glerks are yellow. The island has different kinds of trees. It’s unsafe for Glerks to climb purple trees because the branches on the purple trees break easily.

Judgment DVs (S3, all generations)
forbidden: It is forbidden for Glerks to climb purple trees.
rule: Glerks have a rule against climbing purple trees.
permitted: Glerks are permitted to climb purple trees.
allowed: Glerks are allowed to climb purple trees.
trouble: Glerks get in trouble for climbing purple trees.
punish: Glerks are punished for climbing purple trees.
unsafe: It is unsafe for Glerks to climb purple trees.

Study 1 (“Sleep”)
Seed text (S4)
On the Island of Cloud Hollow, there are two kinds of groups, “Hibbles,” and “Glerks”. Hibbles are blue creatures. Glerks are yellow. The island has different kinds of bushes. It’s unsafe for Glerks to sleep next to purple bushes because snakes live in purple bushes.

Judgment DVs (S4)
forbidden: It is forbidden for Glerks to sleep next to purple bushes.
rule: Glerks have a rule against sleeping next to purple bushes.
permitted: Glerks are permitted to sleep next to purple bushes.
allowed: Glerks are allowed to sleep next to purple bushes.
trouble: Glerks get in trouble for sleeping next to purple bushes.
punish: Glerks are punished for sleeping next to purple bushes.
unsafe: It is unsafe for Glerks to sleep next to purple bushes.

Study 1 (“Explore”)
Seed text (S5)
On the Island of Cloud Hollow, there are two kinds of groups, “Hibbles,” and “Glerks”. Hibbles are blue creatures. Glerks are yellow. The island has different kinds of caves. It’s unsafe for Glerks to go into purple caves because tigers live in purple caves.

Judgment DVs (S5)
forbidden: It is forbidden for Glerks to go into purple caves.
rule: Glerks have a rule against going into purple caves.
permitted: Glerks are permitted to go into purple caves.
allowed: Glerks are allowed to go into purple caves.
trouble: Glerks get in trouble for going into purple caves.

punish: Glerks are punished for going into purple caves.
unsafe: It is unsafe for Glerks to go into purple caves.

Results

Open responses (Scenario-by-Scenario). To analyze scenario-by-scenario trends in the open response scores, we used a mixed-effects logistic regression model that also included a random slope term. The model syntax in R was as follows:

score ~ DV type * Generation + (1 + DV type * Generation | Scenario)

Figure S3 shows the fitted model coefficients for the *DV type* x *generation* term for each scenario. As shown, all estimated coefficients are directionally consistent with the overall results, and no 95% CIs contain $b_{\text{DV type x generation}} = 0$.

Open Responses (Sub-categories). Among the Directive responses, we also analyzed responses that gave *imperatives* and responses that gave *normative guidance* as distinct sub-categories (see "Coding for open responses," main text). This yielded the following categories of interest:

Bare Imperative = responses that relayed an imperative, but not normative guidance nor a descriptive fact, were given a score = 1; score = 0 otherwise.

Imperative+Descriptive = responses that relayed an imperative and a descriptive fact, but not normative guidance, were given a score = 1; score = 0 otherwise.

Bare Normative = responses that relayed normative guidance, but not an imperative nor a descriptive fact, were given a score = 1; score = 0 otherwise.

Normative+Descriptive = responses that relayed normative guidance and a descriptive fact, but not an imperative, were given a score = 1; score = 0 otherwise.

Note the remaining *Directive* responses were "Mixed" in the sense they conveyed *both* an imperative and normative guidance. Since rates of Mixed responses were low (see Figure S4), we did not analyze this category of response.

To analyze responses that gave imperatives, we used a mixed-effects logistic regression model that included fixed effects for *generation* (1, 2, 3, 4) and *DV type* (coded: *Bare Imperative* = 0, *Imperative+Descriptive* = 1), and we included a random intercept term for *scenario*. The model syntax in R was as follows:

score ~ DV type * Generation + (1|Scenario)

For responses that gave normative guidance, we used the same model syntax, with *DV type* coded as *Bare Normative* = 0, *Normative+Descriptive* = 1.

Across generations, the rates of *Bare Imperative* responses increased relative to *Imperative+Descriptive* responses (b = 0.39, se = 0.08, p < .001), as did *Bare Normative* responses relative to *Normative+Descriptive* responses (b = 0.66, se = 0.16, p < .001) (see Figure S4).

Open Responses (Content summaries: Normative responses). To give a better sense of the content of participants' responses, and how the content changed across generations, we report a Table S1 with counts of the types of normative guidance that we coded in Study 1.

Judgments (Overall). To analyze overall trends in judgment ratings, we used a mixed-effects linear regression model that included a fixed effect term for *generation* and a random intercept term for *scenario*. The model syntax in R was as follows:

rating ~ Generation + (1 | Scenario)

Judgments (Scenario-by-Scenario). To analyze scenario-by-scenario trends in the judgment ratings, we used a mixed-effects linear regression model that also included a random slope term:

rating ~ Generation + (1 + Generation | Scenario)

Figure S5 shows the fitted model coefficients for the *Generation* term for each scenario. As shown, the vast majority of estimated coefficients are directionally consistent with the overall results, with the exceptions being: *allowed* and *permitted* for "Sleep," and *allowed* for "Explore."

Judgments (Ordinal logit regression). To ensure the results from the linear regression models are robust, we conducted analyses using ordinal logit regression. To match our original analyses, we fit separate cumulative ordinal logistic regression models for each of the dependent variables (*Rule*, *Forbidden*, *Trouble*, *Punish*, *Allowed*, *Permitted*, and *Unsafe*). Each model predicted *rating* responses (1 = strongly disagree to 6 = strongly agree) from *Generation* with random intercepts and slopes for *Generation* by *Scenario* to account for variation across scenarios. The model syntax was as follows:

rating ~ Generation + (1 + Generation | Scenario)

The effect of generation was consistent with our findings from the linear regression models, reported in the main text. Across generations, ratings increased for *Rule* (b = 0.17, se = 0.04, $p < .001$), *Forbidden* (b = 0.23, se = 0.03, $p < .001$), *Trouble* (b = 0.19, se = 0.05, $p < .001$), and *Punish* (b= 0.18, se = 0.04, $p < .001$); while ratings decreased for *Allowed* (b = -0.11, se = 0.05, p = .015) and *Permitted* (b = -0.13, se = 0.04, p = .001). Ratings for *Unsafe* decreased across generations (b = -0.44, se = 0.06, $p < .001$).

Figure S6 shows the fitted model coefficients for the *Generation* term for each scenario. Figure S7 shows the predicted probabilities for each scale rating, across generations 1 and 4, according to the fitted model.

Studies 2-5: The scope of the inadvisable-to-impermissible effect

Study 2: Evaluative status

Materials. Introductory text, summary instructions, and judgment DVs were the same as in Study 1, Scenario 1 ("Eat"). The seed text read as follows (brackets [] indicate study-specific text):

> On the Island of Cloud Hollow, there are two kinds of groups, "Hibbles," and "Glerks". Hibbles are blue creatures. Glerks are yellow. The island has different kinds of berries. [Glerks do not like eating purple berries because they think purple berries are icky].

Results

Open responses. To analyze open response scores, we used a logistic regression model with *DV type* (coded: *Directive* = 0, *Descriptive* = 1) and *generation* as fixed effect terms. The model syntax in R was: score ~ DV type * Generation. For further analysis, we also used the same model specification but included the "fine-grained" coding for *DV type* (coded: *Bare Directive* = 0, *Directive* +*Descriptive* = 1, *Bare Descriptive* = 2).

Judgments (overall; linear regression). To analyze judgment ratings, we used a linear regression model *generation* as a fixed effect term. The model syntax in R was: rating ~ Generation. Results are reported in the main text.

Judgments (overall; ordinal logistic regression). We fit separate cumulative ordinal logistic regression models for each of the dependent variables (*Rule*, *Forbidden*, *Trouble*, *Punish*, *Allowed*, *Permitted*, and *Unsafe*). Each model predicted *rating* responses (1 = strongly disagree to 6 = strongly agree) from *Generation.* The model syntax in R was: rating ~ Generation.

As we found with the linear regression models, ratings for prohibition and punishment judgments increased across generations (*Rule*: b = 0.17, se = 0.03, p < .001; *Forbidden*: b = 0.22, se = 0.03, p < .001; *Trouble*: b = 0.19, se = 0.03, p < .001; *Punish*: b = 0.19, se = 0.03, p < .001), and ratings for permission judgments decreased across generations (*Permitted*: b = -0.13, se = 0.03, p < .001; *Allowed*: b = -0.11, se = 0.03, p = .001). Ratings for *Unsafe* also increased across generations (b = -0.42, se = 0.05, p < .001). See Figure S8 for a visual depiction.

Judgments (by response type received). To analyze judgment ratings according to response type received, we used a linear regression model with response *type* received as a fixed effect term (coded, Model 1: *Directive+Descriptive* = 0, *Bare Directive* = 1; coded, Model 2: *Bare Descriptive* = 0, *Directive +Descriptive* = 1). The model syntax in R was: rating ~ type.

Study 3: Subjects

Materials. Introductory text, summary instructions, and judgment DVs were the same as in Study 1, Scenario 1 (“Eat”). The seed text read as follows:

> On the Island of Cloud Hollow, there are two kinds of groups, “Hibbles,” and “Glerks”. Hibbles are blue creatures. Glerks are yellow. The island has different kinds of [berries. It's unsafe for Hibbles to eat purple berries because purple berries are poisonous to Hibbles].

Results

Open responses. To analyze open response scores, we used the same model specifications as Study 2, though *DV type* was coded as *Bare Directive: patient* = 0, *Bare Directive: subject* = 1.

Judgments (linear regression). To analyze judgment ratings, we used the same model specifications as Study 2. Results are reported in the main text.

Judgments (ordinal logistic regression). For supplemental analysis, we used the same ordinal logistic regression model specifications as Study 2. The results were consistent with those from the linear regression model (see Figure S9). Across generations, ratings increased for *Rule* (b = 0.71, se = 0.10, p < .001), *Forbidden* (b = 0.67, se = 0.10, p < .001), *Trouble* (b = 0.68, se = 0.`0, p < .001), and *Punish* (b= 0.58, se = 0.10, p < .001); while ratings decreased for *Allowed* (b = -0.52, se = 0.10, p < .001) and *Permitted* (b = -0.53, se = 0.10, p < .001). Ratings for *Unsafe* decreased across generations (b = 0.72, se = 0.10, p < .001).

Study 4: Norm maintenance

Materials. Introductory text, summary instructions, and judgment DVs were the same as in Study 1, Scenario 1 (“Eat”). The seed text read as follows:

> On the Island of Cloud Hollow, there are two kinds of groups, “Hibbles,” and “Glerks”. Hibbles are blue creatures. Glerks are yellow. The island has different kinds of [berries. Glerks are not allowed to eat purple berries].

Results

Open responses. To analyze open response scores, we used the same model specifications as Study 2.

Judgments (linear regression). To analyze judgment ratings, we used the same model specifications as Study 2. Results are reported in the main text.

Judgments (ordinal logistic regression). For supplemental analysis, we used the same ordinal logistic regression model specifications as Study 2. The results were consistent with those from the linear regression model (see Figure S10). Across generations, ratings did not change for *Rule* (b = 0.10, se = 0.10, p = .31), *Trouble* (b = 0.06, se = 0.09, p = .51), and *Punish* (b= 0.07, se = 0.09, p = .45); while ratings decreased for), *Forbidden* (b = -0.25, se = 0.10, p = .01) and increased for *Allowed* (b = 0.44, se = 0.12, p < .001) and *Permitted* (b = 0.33, se = 0.11, p = .004). Ratings for *Unsafe* increased across generations (b = 0.37, se = 0.10, p < .001).

Study 5: Horizontal transmission

Materials. Introductory text, seed text, and judgment DVs were the same as in Study 1, Scenario 1 ("Eat"). The summary instructions were as follows: "Imagine you are a Glerk, and you're meeting a new Glerk who has just moved to the island. What would you say to teach the new Glerk about the island?"

Results

Open responses. To analyze open response scores, we used the same model specifications as Study 2.

Judgments (linear regression). To analyze judgment ratings, we used the same model specifications as Study 2. Results are reported in the main text.

Judgments (ordinal logistic regression). For supplemental analysis, we used the same ordinal logistic regression model specifications as Study 2. The results were consistent with those from the linear regression model (see Figure S11). Across generations, ratings increased for *Rule* (b = 0.23, se = 0.10, p = .01), *Forbidden* (b = 0.23, se = 0.09, p = .01), *Trouble* (b = 0.32, se = 0.09, p < .001), and *Punish* (b= 0.26, se = 0.09, p = .006); while ratings did not change for *Allowed* (b = -0.07, se = 0.10, p = .45) and *Permitted* (b = -0.09, se = 0.10, p = .37). Ratings for *Unsafe* decreased across generations (b = -0.62, se = 0.15, p < .001).

Introduction text (generations 0-1):	Introduction text (generations 2-4):
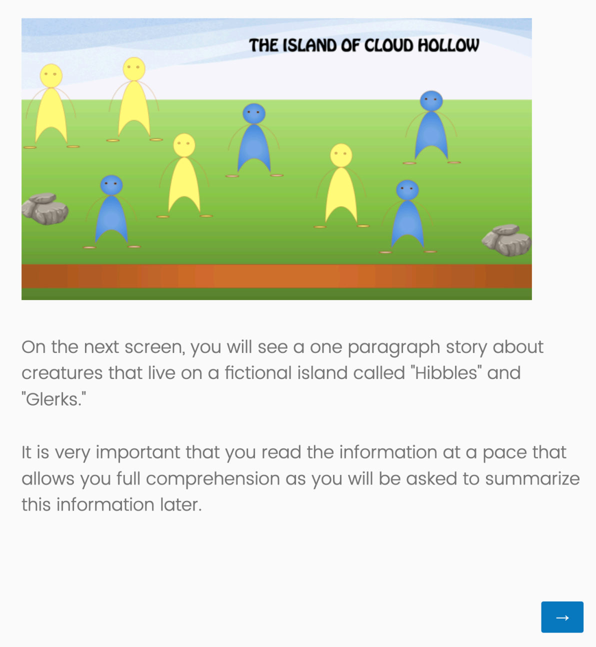	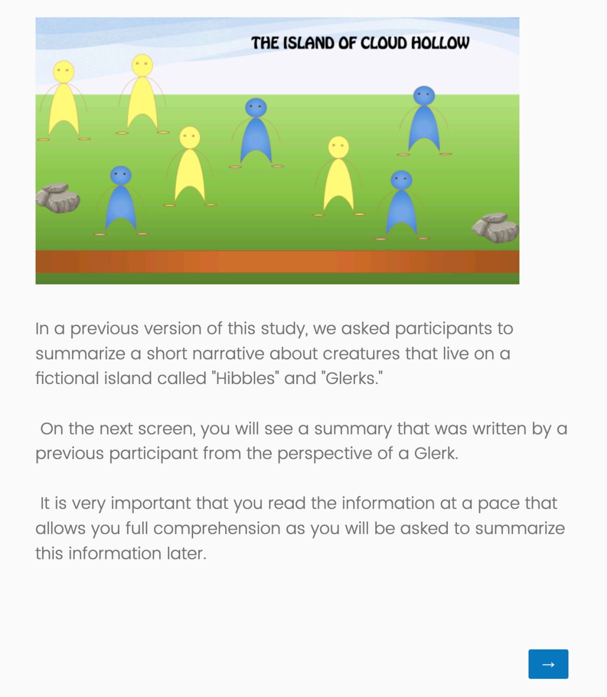

Fig. S1. Introduction text viewed by participants.

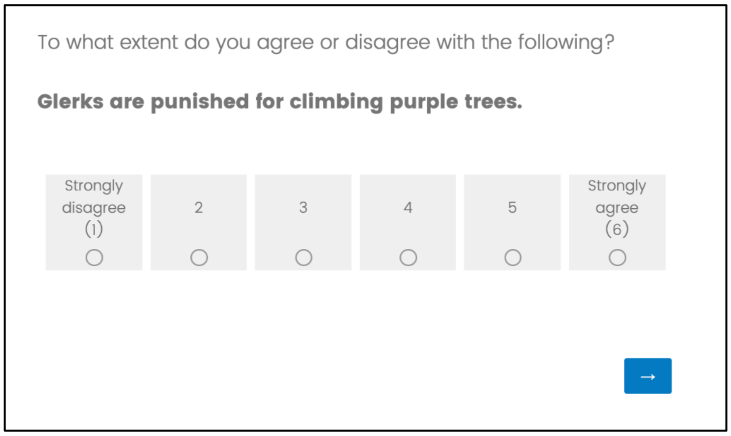

Fig. S2. Example of judgment DV as viewed by participants.

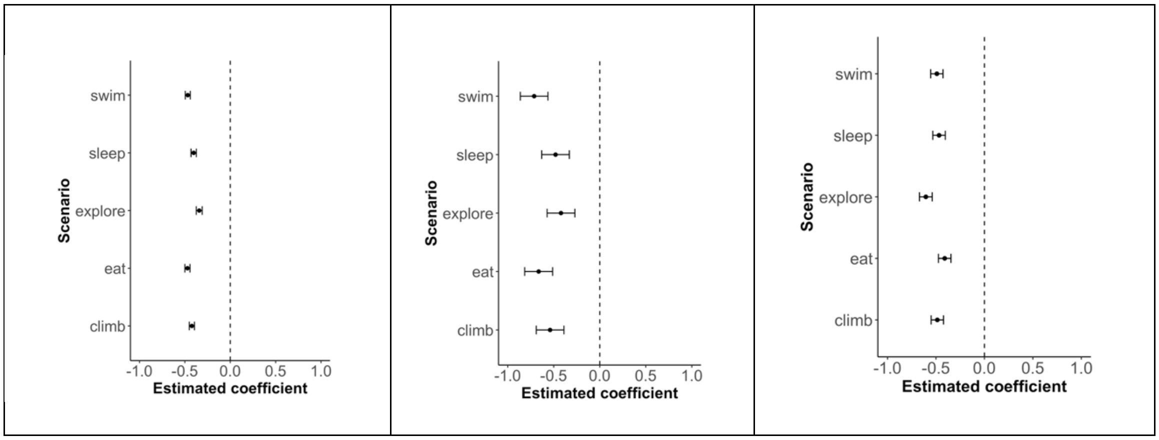

Fig. S3. (Left) Estimated coefficients for the *DV type* x *generation* term, where *DV type* ∈ {*Directive, Descriptive*}. Error bars correspond to 95% CIs. Dashed line indicates estimated coefficient $b_{\text{DV type x generation}}$ = 0. (Center) Estimated coefficients for the *DV type* = *Directive+Descriptive* x *generation* term, where *DV type* ∈ {*Bare Deontic, Directive+Descriptive, Bare Descriptive*}. (Right) Estimated coefficients for the *DV type* = *Bare Descriptive* x *Generation* term.

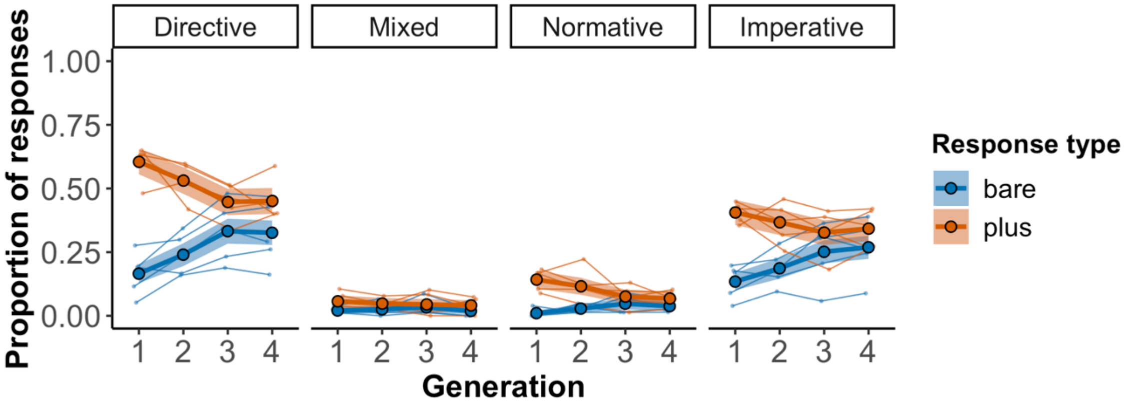

Fig. S4. Proportion of responses conveying each type of utterance. “Bare” responses (blue) do not contain descriptive information. “Plus” responses (orange) contain descriptive information. The thick lines with large dots correspond to the mean proportion across all five studies, with shaded regions corresponding to 95% CIs. The thin lines correspond to the mean proportion from the individual scenarios. *Directive* (far left panel) is the coding for analyses in the main text. The category of *Directive* is comprised of (i) *Mixed* responses conveying both an imperative and normative guidance, (ii) *Normative* responses that convey normative guidance, but not an imperative, and (iii) *Imperative* responses that convey an imperative, but not normative guidance.

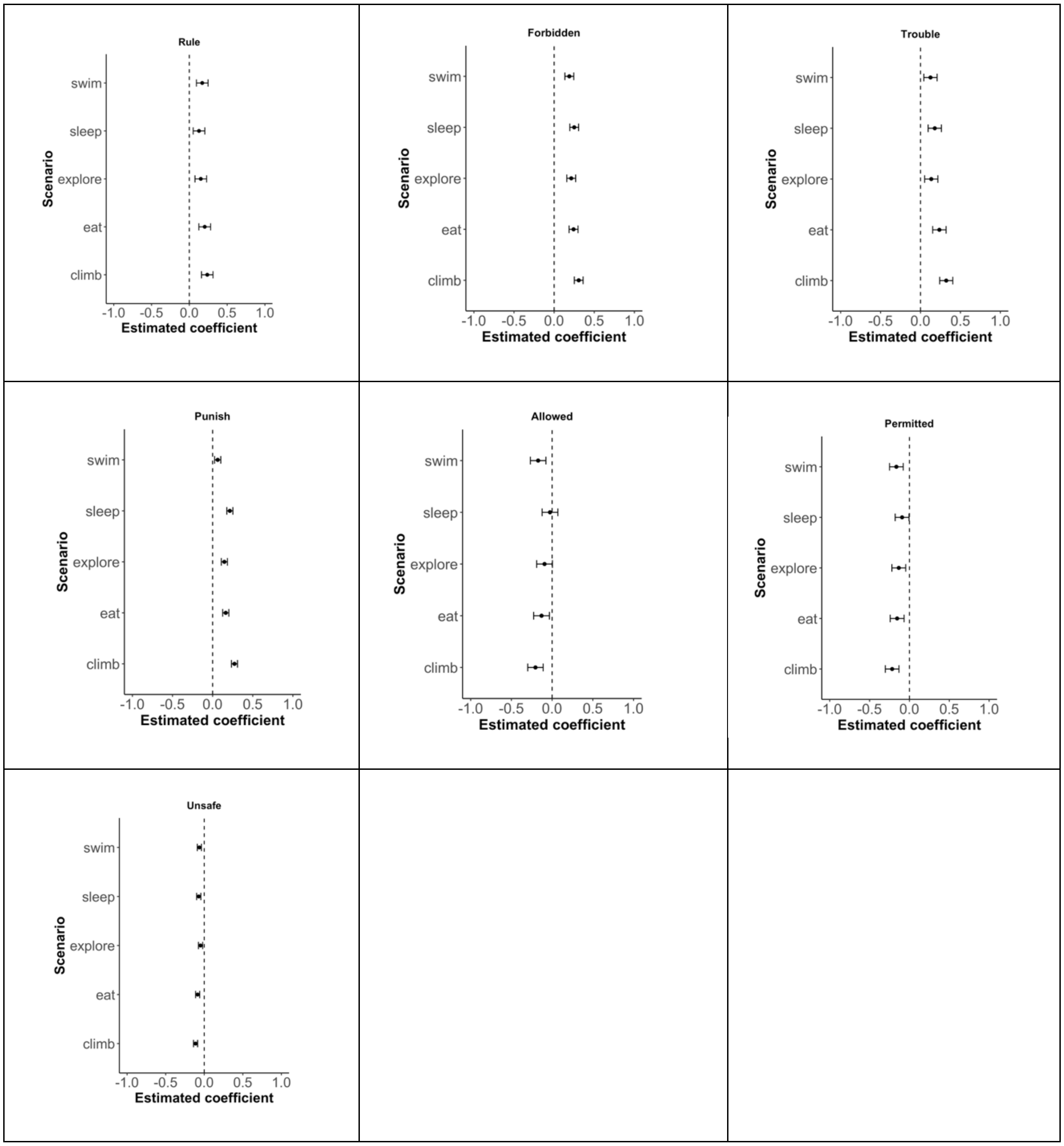

Fig. S5. Study 1, linear regression: Estimated coefficients (x-axis) for the *generation* term, for each scenario (y-axis). Error bars correspond to 95% CIs. Dashed line indicates estimated coefficient $b_{\text{generation}} = 0$.

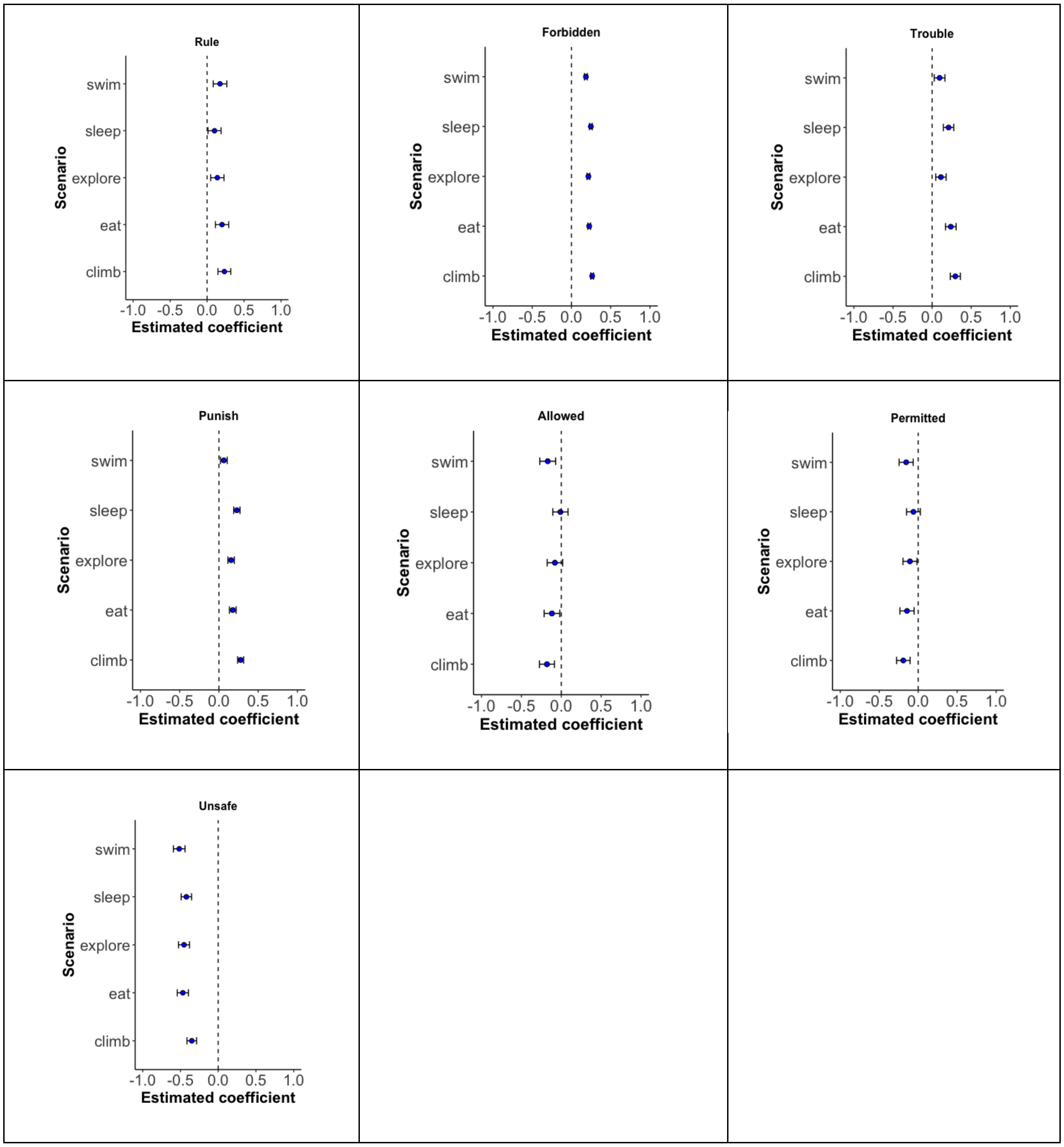

Fig. S6. Study 1, ordinal logistic regression: Estimated coefficients (x-axis) for the *generation* term, for each scenario (y-axis). Error bars correspond to 95% CIs. Dashed line indicates estimated coefficient $b_{\text{generation}} = 0$.

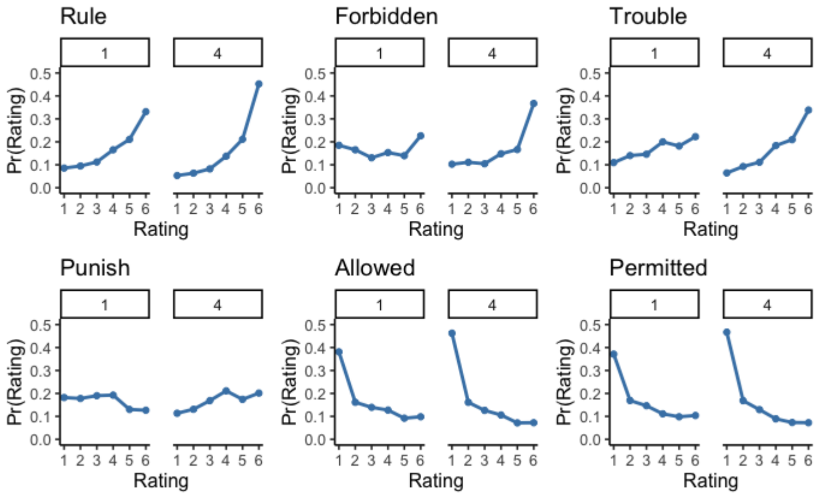

Fig. S7. Study 1: predicted probability (y-axis) for each scale rating (x-axis), according to the fitted ordinal logistic regression model (left panels: Generation 1; right panels: Generation 4).

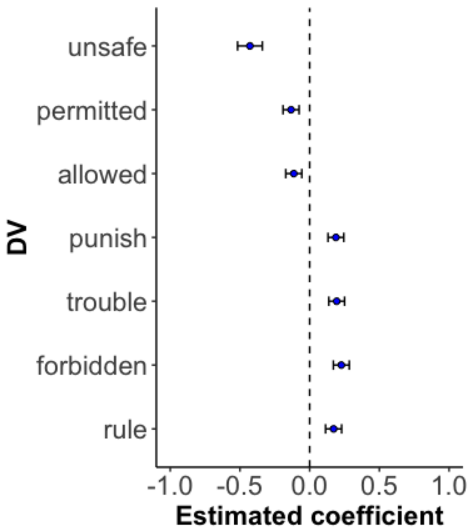

Fig. S8. Study 2, ordinal logistic regression: Estimated coefficients (x-axis) for the *generation* term, for each DV (y-axis). Error bars correspond to 95% CIs. Dashed line indicates estimated coefficient $b_{\text{generation}} = 0$.

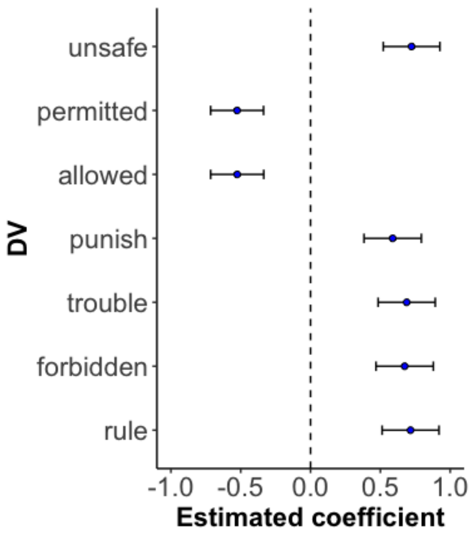

Fig. S9. Study 3, ordinal logistic regression: Estimated coefficients (x-axis) for the *generation* term, for each DV (y-axis). Error bars correspond to 95% CIs. Dashed line indicates estimated coefficient $b_{generation} = 0$.

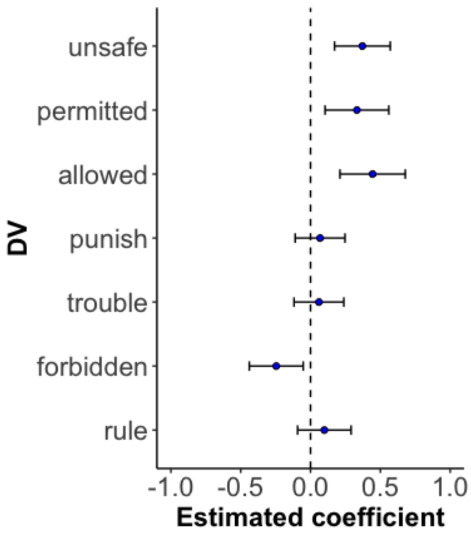

Fig. S10. Study 4, ordinal logistic regression: Estimated coefficients (x-axis) for the *generation* term, for each DV (y-axis). Error bars correspond to 95% CIs. Dashed line indicates estimated coefficient $b_{\text{generation}} = 0$.

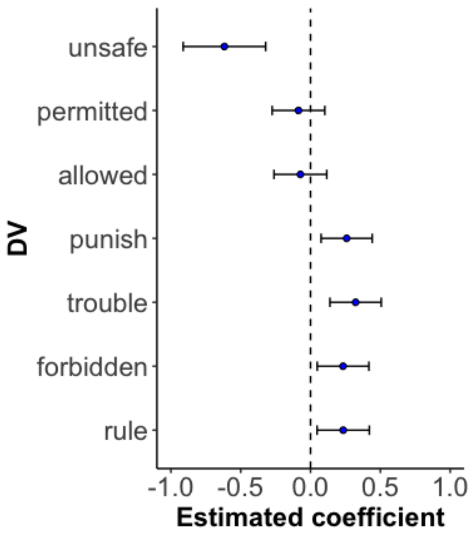

Fig. S11. Study 5, ordinal logistic regression: Estimated coefficients (x-axis) for the *generation* term, for each DV (y-axis). Error bars correspond to 95% CIs. Dashed line indicates estimated coefficient $b_{generation} = 0$.

Table S1. Counts for type of normative guidance in responses from Study 1

	**Generation**			
**Content**	**1**	**2**	**3**	**4**
“cannot”	22	4	4	6
“follow the rule”	0	1	1	1
“forbidden”	0	1	0	0
“have to”	0	2	2	1
“must”	7	7	10	10
“need to”	4	5	4	1
“never”	45	38	34	32
“not allowed to”	2	2	0	2
“not supposed to”	0	0	0	1
“only”	0	1	1	0
“should”	4	13	11	5
“should not”	5	5	3	4
